# Clauson–Kaas pyrrole synthesis using diverse catalysts: a transition from conventional to greener approach

**DOI:** 10.3762/bjoc.19.71

**Published:** 2023-06-27

**Authors:** Dileep Kumar Singh, Rajesh Kumar

**Affiliations:** 1 Department of Chemistry, Bipin Bihari College, Affiliated to Bundelkhand University, Jhansi-284001, Uttar Pradesh, India; 2 P.G. Department of Chemistry, R. D. S. College, B. R. A. Bihar University, Muzaffarpur-842002, Bihar, India

**Keywords:** catalyst, Clauson–Kaas pyrrole synthesis, 2,5-dimethoxytetrahydrofuran, green synthesis, microwave-assisted reaction, N-substituted pyrrole

## Abstract

Pyrrole is an important aromatic heterocyclic scaffold found in many natural products and predominantly used in pharmaceuticals. Continuous efforts are being made to design and synthesize various pyrrole derivatives using different synthetic procedures. Among them, the Clauson–Kaas reaction is a very old and well-known method for synthesizing a large number of N-substituted pyrroles. In recent years, due to global warming and environmental concern, research laboratories and pharmaceutical industries around the world are searching for more environmentally friendly reaction conditions for synthesizing compounds. As a result, this review describes the use of various eco-friendly greener protocols to synthesize N-substituted pyrroles. This synthesis involves the reaction of various aliphatic/aromatic primary amines, and sulfonyl primary amines with 2,5-dimethoxytetrahydrofuran in the presence of numerous acid catalysts and transition metal catalysts. The goal of this review is to summarize the synthesis of various N-substituted pyrrole derivatives using a modified Clauson–Kaas reaction under diverse conventional and greener reaction conditions.

## Introduction

**Figure 1:**
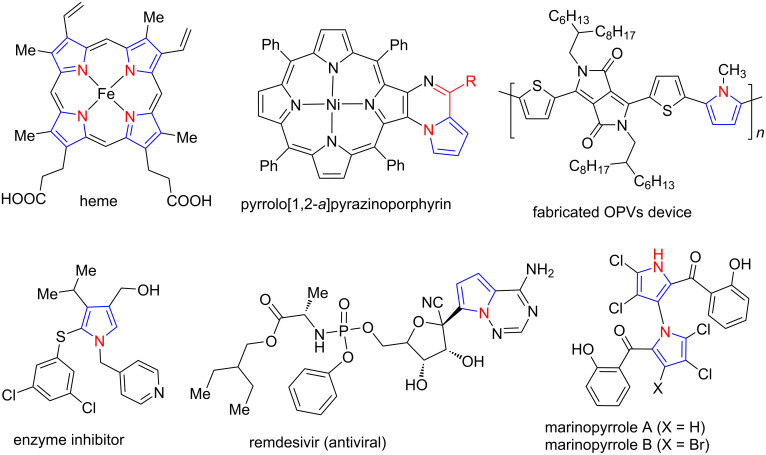
Various pyrrole containing molecules.

Heterocyclic compounds are the most explored molecules in organic chemistry in terms of their synthesis and various applications. Among the diverse heterocyclic compounds, N-containing heterocycles are found in many natural products [1–3] and biologically active molecules [4–7]. Pyrroles are a significant class of five-membered aromatic nitrogen-containing heterocyclic skeletons that have attracted much attention due to their broad spectrum of biological activity, such as anticancer [8–10], antiviral [11–12], antibacterial [13–15], antimalarial [16–17], anti-inflammatory [18–19], anti-oxidant [20–22], antifungal [23–24] and antibiotic [25–26] and as enzyme inhibitors [27–28]. Several pharmaceuticals, polymers and naturally occurring compounds, including heme, chlorophyll, vitamin B12, porphyrins, chlorins, and bacteriochlorins also contain pyrroles rings (Figure 1).

**Scheme 1:**
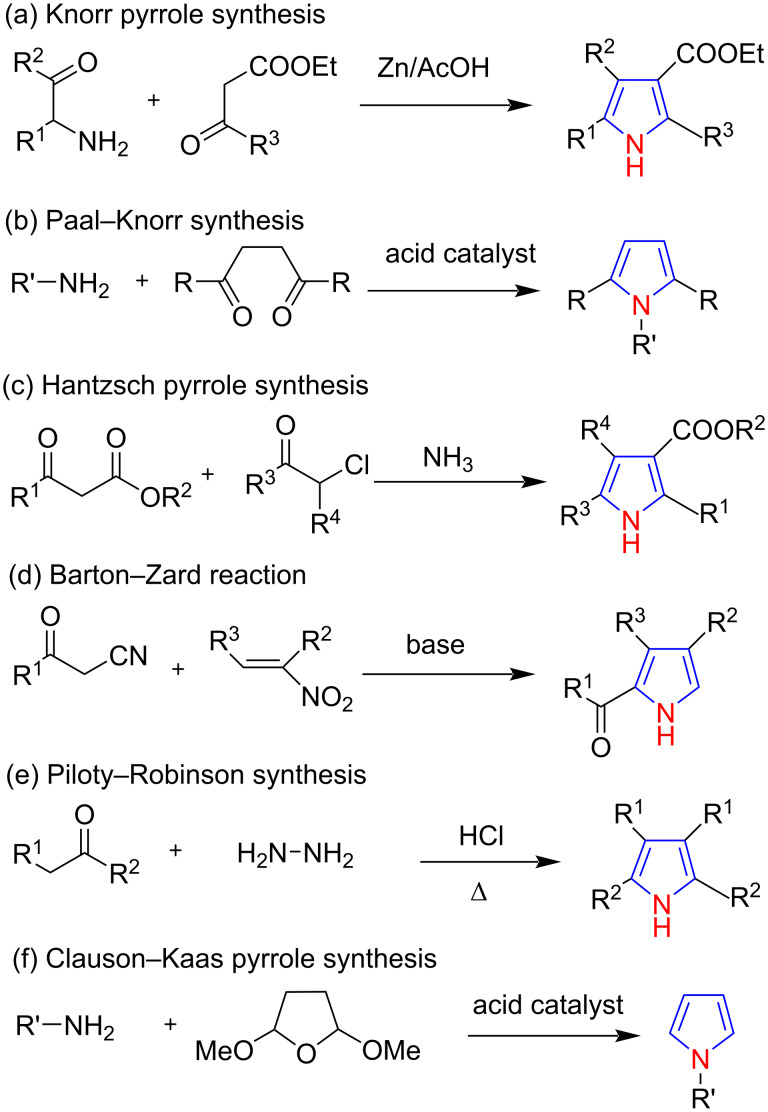
Various synthestic protocols for the synthesis of pyrroles.

For the synthesis of pyrrole derivatives, many classic methods have been used, including Knorr pyrrole synthesis [29–30], Paal–Knorr synthesis [31–33], Hantzsch pyrrole synthesis [34–36], Clauson–Kaas synthesis [37–38], Piloty–Robinson synthesis [39–41], and Barton–Zard reaction [42–44] (Scheme 1). Among these, the Clauson–Kaas pyrrole synthesis has received much attention because the synthesized pyrrole contains unsubstituted carbons that can be used for further functionalization. This methodology has been applied to diverse areas of chemistry, including natural product synthesis [45–46], medicinal chemistry [47–48], polymer chemistry [49–50] and porphyrin chemistry [51–53].

**Figure 2:**
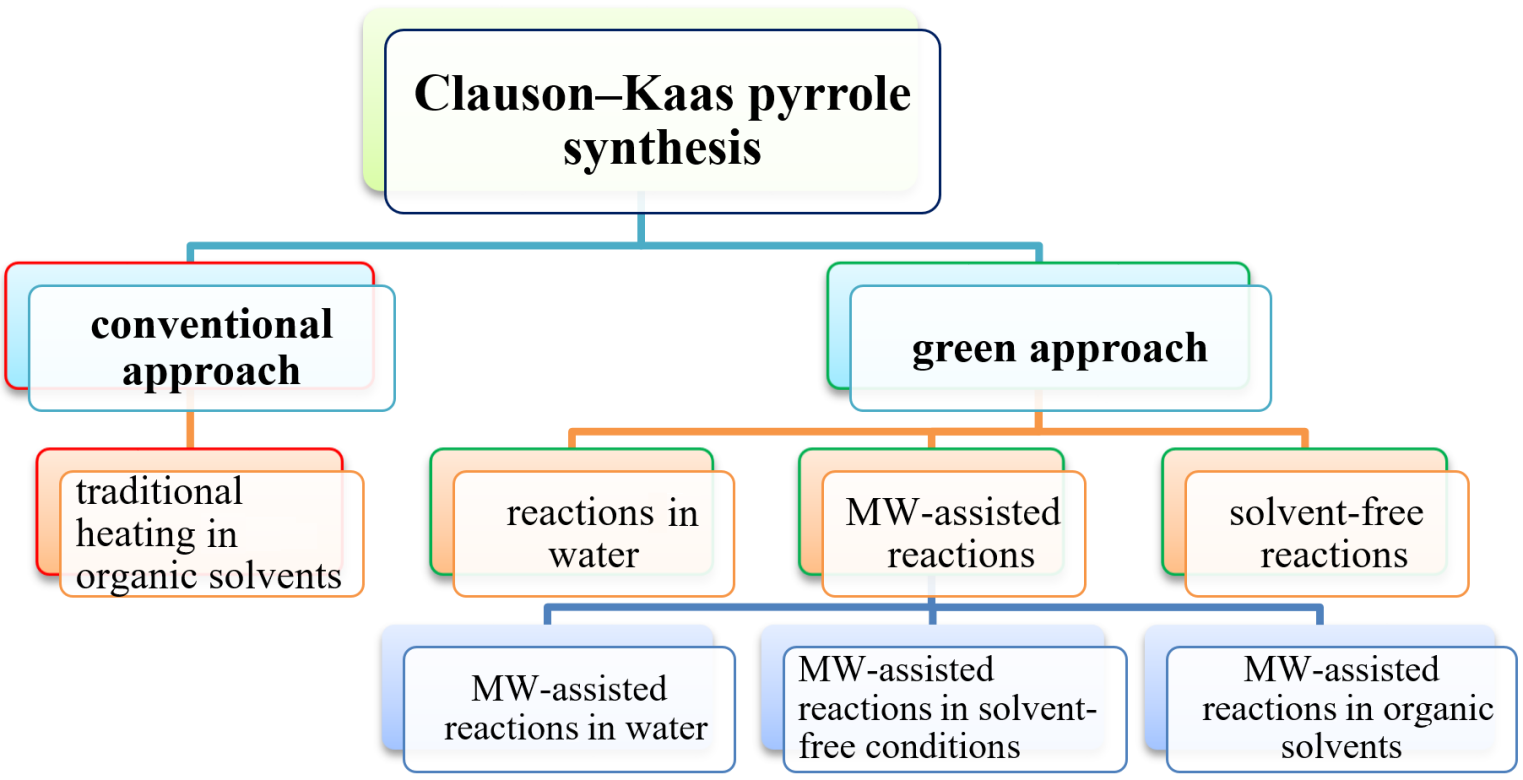
A tree-diagram showing various conventional and green protocols for Clauson-Kaas pyrrole synthesis.

In recent years, green chemistry has become a widely used method for organic synthesis in order to reduce energy consumption and the carbon footprint. The traditional heating techniques used to synthesize compounds are being replaced by contemporary green alternative energy systems, such as photocatalysis, microwave irradiation, ultrasonic irradiation, grinding, and ball milling processes. Furthermore, many solvent-free reactions and solid-supported reagents are becoming increasingly popular in organic synthesis. Moreover, in order to increase the product yield, different green catalysts were used in the synthesis of numerous organic and bioorganic molecules while reducing the amount of excess solvent and reaction time. The environmental impacts are manifold, including excessive utilization of organic solvents, high-temperature reactions, the production of hazardous byproducts, and by the use of traditional purification techniques. Therefore, green chemistry methods in Clauson–Kaas synthesis are desired in light of growing environmental concerns. As a result, this review will explain various green chemistry approaches to synthesize N-substituted pyrroles. Although, Wynn [54] summarized several Clauson–Kaas protocols for the synthesis of N-substituted pyrrole derivatives. However, a comprehensive review on Clauson–Kaas pyrrole synthesis has not yet been published. Therefore, a review on this topic was required, describing various reaction conditions used in the Clauson–Kaas reaction. At the end, this review is divided into two sections. The conventional Clauson-Kaas pyrrole synthesis procedure is explained in the first section, and the green protocol is covered in the second (Figure 2).

The second section is further divided into three subsections that covers the various eco-friendly green processes used, such as, (i) reactions in water, (ii) solvent-free reactions, (iii) microwave-assisted reactions in water, solvent-free conditions and in other organic solvents.

### Clauson–Kaas reaction and its mechanism

**Scheme 2:**
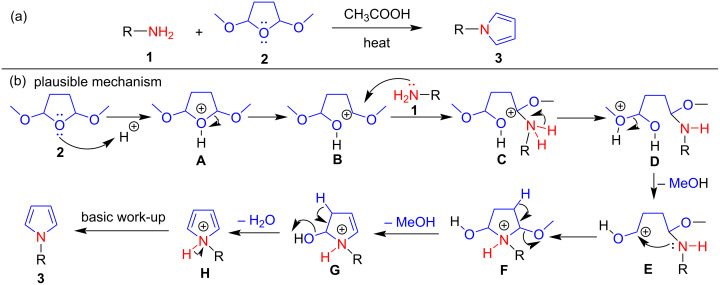
A general reaction of Clauson–Kaas pyrrole synthesis and proposed mechanism.

The Clauson–Kaas reaction refers to the synthesis of various N-substituted pyrroles via an acid-catalyzed reaction between aromatic or aliphatic primary amines and 2,5-dialkoxytetrahydrofuran. This reaction was originally discovered by N. Clauson–Kaas and Z. Tyle in 1952 [37] (Scheme 2a). Initially, acetic acid was used as a catalyst in this classic reaction; however, diverse modifications have been reported for this procedure using various Brønsted acid catalysts, metal catalysts, and nanoorganocatalysts. Various solvent systems, such as aqueous conditions, different organic solvents, solvent-free conditions, ionic liquids, and DESs, have been reported in modified Clauson–Kaas reactions at room temperature, under thermal and microwave-assisted conditions. In the Clauson–Kaas reaction mechanism proposed by Wang [55] (Scheme 2b), 2,5-dimethoxytetrahydrofuran (**2**) is first protonated with acetic acid, followed by ring opening to form carbocation **B**. In the following step, primary amine **1** nucleophilically attacks carbocation **B** to produce intermediate **C**, which, after proton rearrangement and the removal of methanol, produces intermediate **E**.

In the next steps, the ring closure of intermediate **E** due to the lone pair of nitrogen gives **F**, which after the elimination of methanol and water in subsequent steps provides intermediate **H**. Finally, an aromatic N-substituted pyrrole **3** is produced by basic work-up of intermediate **H** (Scheme 2b).

## Review

### Conventional method for the Clauson–Kaas synthesis of N-substituted pyrroles

**Scheme 3:**
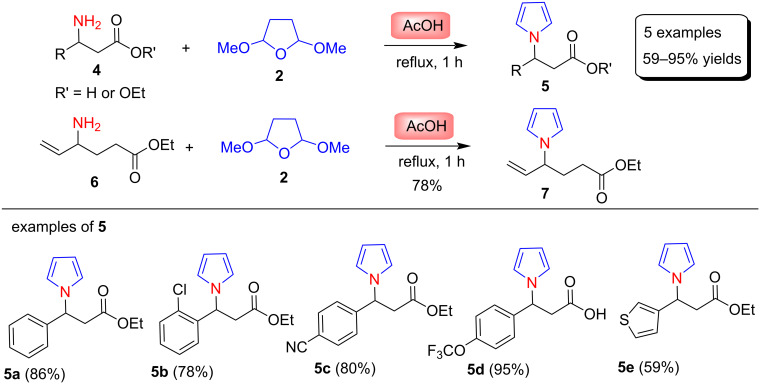
AcOH-catalyzed synthesis of pyrroles **5** and **7**.

This section describes Clauson–Kaas pyrrole syntheses using traditional methods, such as Brønsted acid or Lewis acid-catalyzed reactions in various organic solvents at higher temeperatures. In 2000, the application of the Clauson–Kaas reaction was nicely explored by Sonnet et al. [56] for the preparation of diverse pyrrolizinones. The pyrrole derivatives **5** and **7** were synthesized by the classical Clauson–Kaas reaction by refluxing amines **4** or **6** with 2,5-dimethoxytetrahydrofuran (**2**) in acetic acid, affording ethyl arylpyrrolylpropionates **5** in 59–95% and pyrrole **7** in 78% yield. Representative examples of compounds **5** are shown in Scheme 3. Furthermore, these N-substituted pyrroles were used to prepare various aryl-substituted pyrrolizine and indolizine derivatives.

**Scheme 4:**
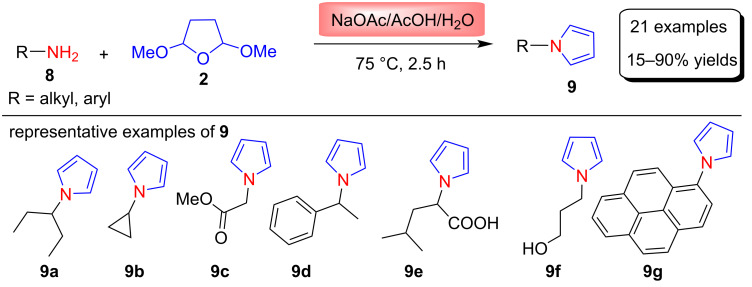
Synthesis of N-substituted pyrroles **9**.

In 2014, Kumar and co-workers [57] synthesized various N-substituted pyrrole derivatives **9** using the Clauson–Kaas reaction to study their structure–reactivity relationship (SRR). These compounds were synthesized in 15–90% yields from the reaction between various aliphatic and aromatic amines **8** and 2,5-DMTHF (**2**) in the presence of NaOAc under an AcOH/H_2_O mixture at 75 °C in 2.5 h (Scheme 4). Further, these synthesized N-α-substituted compounds were subjected to electropolymerization studies.

**Scheme 5:**
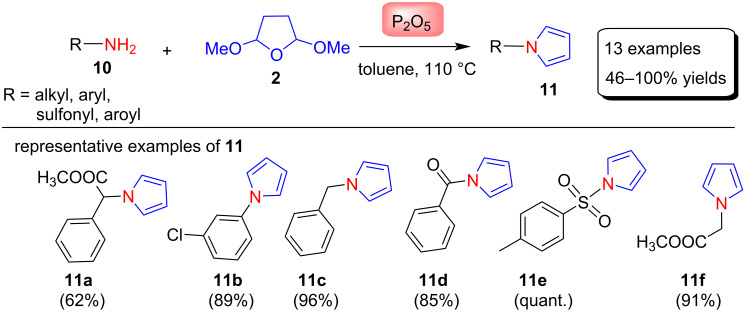
P_2_O_5_-catalyzed synthesis of N-substituted pyrroles **11**.

In another method described for the synthesis of N-substituted pyrroles **11**, Fang et al. [58] used phosphorus pentoxide (P_2_O_5_) as a catalyst for the conversion of various aliphatic amines, aromatic amines, sulfonamides and primary amides into N-substituted pyrroles (Scheme 5). These pyrroles were synthesized in 46–100% yields by the modified Clauson–Kaas reaction between amines **10** and 2,5-DMTHF (**2**) in the presence of P_2_O_5_ under toluene at 110 °C. Since phosphorus pentoxide gives phosphoric acid esters upon reaction with alcohols and also has less acidic character, the authors hypothesized that it might be a good choice for the conversion of amines **10** into their corresponding pyrroles. The results were according to their expectation thus, they synthesized 13 derivatives of pyrroles in good yields in short reaction times (10–45 min). It has been observed that aromatic amines and amides take a longer time compared to the primary amines and sulfonamides.

**Scheme 6:**
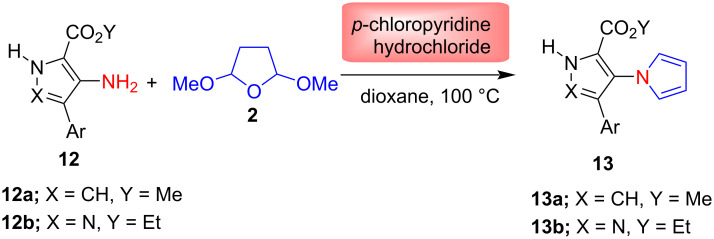
*p*-Chloropyridine hydrochloride-catalyzed synthesis of pyrroles **13**.

In another study, Rochais et al*.* [59] reported in 2004 the synthesis of arylpyrrolo- and pyrazolopyrrolizinones, in which the Clauson–Kaas reaction was used as key step for the preparation of pyrrole derivatives **13**. The condensation of amines **12** with 2,5-DMTHF (**2**) was carried out in the presence of *p*-chloropyridine hydrochloride as catalyst and dioxane as reaction solvent at 100 °C (Scheme 6). Finally, compounds **13** were used to prepare various arylpyrrolo- and pyrazolopyrrolizinones for diverse biological activity.

**Scheme 7:**
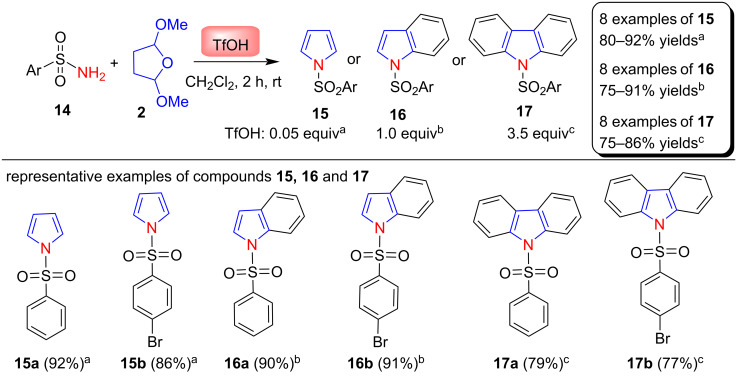
TfOH-catalyzed synthesis of *N*-sulfonylpyrroles **15**, *N*-sulfonylindole **16, ***N*-sulfonylcarbazole **17**.

In 2007, Török and co-workers [60] reported a convenient one-pot synthesis of N-sulfonyl-substituted pyrroles, indoles, and carbazole via a modified Clauson–Kaas reaction in a successive cyclization/annulation process from commercially available sulfonamides **14** in the presence of trifluomethanesulfonic acid (TfOH) as Brønsted-acid catalyst. This procedure produces only N-substituted products and preserves other positions open for further functionalization. The formation of the desired products depends on the amount of triflic acid used in the reaction. Using 0.05 equivalents triflic acid gives *N*-sulfonylpyrroles **15** in 80–92% yields, 1.0 equivalent of triflic acid provides N-sulfonylindole **16** in 75–91% yields and 3.5 equivalents of triflic acid gives *N*-sulfonylcarbazole **17** in 75–86% yields as shown in Scheme 7. This procedure provides the selectivities in a very short time and gives products in excellent yields.

**Scheme 8:**
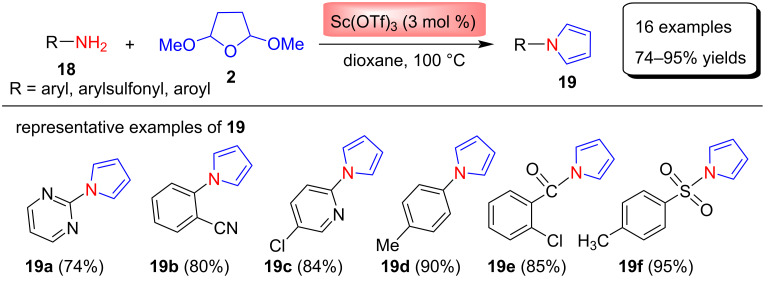
Scandium triflate-catalyzed synthesis of N-substituted pyrroles **19**.

Zuo et al. [61] reported the Clauson–Kaas synthesis of N-substituted pyrroles **19** using scandium triflate as the catalyst in good to excellent yields. To obtain the best reaction conditions, various Lewis acid catalysts (e.g., FeCl_3_ CuCl_2_, InCl_3_, Cu(OTf)_2_, Mg(OTf)_2_, Zn(OTf)_2_, Yb(OTf)_3_, Y(OTf)_3_, Bi(OTf)_3_, La(OTf)_3_ and Sc(OTf)_3_), different solvents (e.g., CH_2_Cl_2_, CHCl_3_, CH_3_CN, CH_3_NO_2_, *n*-hexane, and dioxane), temperatures (90–110 °C), and the catalyst loadings have been investigated. After optimizing the reaction conditions, 3 mol % of Sc(OTf)_3_ as catalyst, 1,4-dioxane as solvent, and a temperature of 100 °C were selected. Finally, using these reaction conditions, 16 examples of N-substituted pyrroles were synthesized by the reaction between various aromatic, sulfonyl- and aroylamines **18** with 2,5-DMTHF (**2**) in yields of 74–95% as shown in Scheme 8. Among various methods available for the synthesis of N-substituted pyrroles, this protocol requires less reaction time, mild reaction conditions, easy workup, and provides improved yields of the products. Furthermore, this method shows good functional group tolerance, because various aromatic amines, benzamides, and sulfonamides were successfully used as substrates in these transformations. In this study, it was found that the reactions of aromatic amines bearing electron-withdrawing groups or electron-donating groups proceed smoothly without showing any significant substituent effect. Also, there was no effect of the substituent on yields and reaction time.

**Scheme 9:**
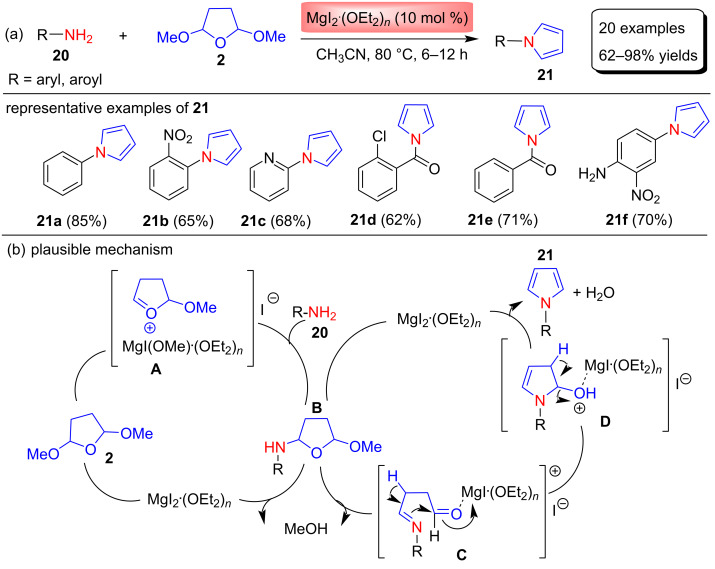
MgI_2_ etherate-catalyzed synthesis and proposed mechanism of *N*-arylpyrrole derivatives **21**.

In 2011, Zhang and Shi [62] reported the Clauson–Kaas synthesis of N-substituted pyrroles **21** through the reaction between various substituted anilines, primary arylamides, and sufonylamides **20** and 2,5-DMTHF (**2**) in the presence of 10 mol % MgI_2_ etherate in MeCN at 80 °C (Scheme 9a). MgI_2_ etherate is a main-group Lewis acid catalyst that selectively activates electron-rich aromatic amines. This is a mild, efficient, and highly chemoselective procedure in which the iodine counterion and MeCN played key roles in the unique reactivity of this catalytic system. To optimize the reaction conditions, many catalysts, solvents, and temperatures were studied and finally, 10 mol % MgI_2_∙(OEt_2_)*_n_* as the catalyst, CH_3_CN as the solvent, and 80 °C were selected and used for the synthesis of 20 examples of N-substituted pyrroles in yields of 62–98%. In this study, it was observed that anilines with electron‐withdrawing groups (i.e., NO_2_, CF_3_, F, Cl, Br) deactivated arylamine and gave the corresponding pyrroles in moderate yields, while anilines with electron‐donating groups (i.e., OMe, Me) reacted much faster than aniline and furnished the pyrroles in excellent yields. In addition, the reaction of more sterically hindered aniline with 2,5-dimethoxytetrahydrofuran gave moderate yields and less sterically hindered aniline gave good yields. This reactivity of the aromatic amine depends on the electron density of the amino compounds.

In addition, the authors also performed the reaction of aliphatic amines with 2,5-DMTHF (**2**), and found that aliphatic amines are inert in the presence of MgI_2_ etherate. The proposed mechanism shown in Scheme 9b suggests the ability of MgI_2_ etherate to act as a Lewis acid activator. The iodine counterion is coordinated to the Lewis basic oxygen atom of the acetal group to give the more Lewis acidic cataonic Mg-coordinated intermediate **A**. Intermediate **A** upon nucleophilic reaction with amines **20** yields **B**, which upon subsequent removal of MeOH, dehydration and aromatization affords N-substituted pyrroles **21**.

**Scheme 10:**
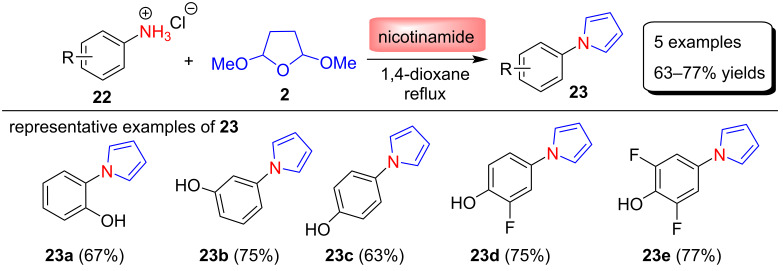
Nicotinamide catalyzed synthesis of pyrroles **23**.

In 2013, Chatzopoulou [63] and co-workers reported a high-yielding Clauson–Kaas pyrrolyl-phenol synthesis using nicotinamide, which is a cheap and nontoxic catalyst and a vitamin and enzyme cofactor. The authors chose nicotinamide as the catalyst for this study because it has a p*K*_a_ of 3.43 and could act as a chemical antioxidant. In some cases, aminophenol hydrochloride is the only form of the corresponding aminophenol that can be isolated, so use of aminophenol hydrochloride as the starting material is also an important aspect of this protocol. The N-substituted pyrrole derivatives **23** were synthesized in 63–77% yields by the Clauson–Kaas reaction between various aminophenol hydrochlorides **22** and 2,5-dimethoxytetrahydrofuran (**2**) in the presence of an equimolar amount of nicotinamide in 1,4-dioxane under reflux conditions (Scheme 10).

### Green chemistry approach for the Clauson–Kaas synthesis of N-substituted pyrroles

This section describes the Clauson-Kaas pyrrole syntheses using different greener methods in the presence of various Brønsted acids or Lewis acids. These include reactions in aqueous media, under solvent-free conditions, and under microwave irradiation.

#### (1) Reactions in water

**Scheme 11:**
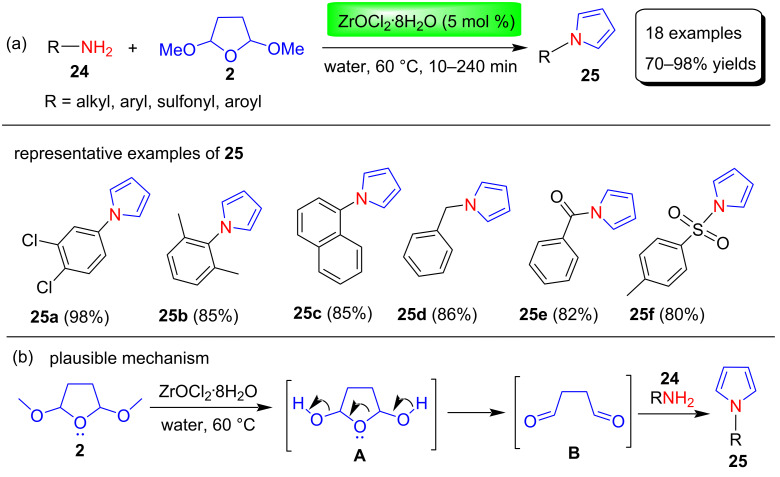
ZrOCl_2_∙8H_2_O catalyzed synthesis and proposed mechanism of pyrrole derivatives **25**.

In recent years, organic reactions in aqueous medium have grown in popularity due to their low cost and environmental friendliness. The use of water as a solvent medium for the synthesis of pyrrole derivatives using various catalysts is described here. Ghafuri and Emami [64] reported a simple, efficient, and green Clauson–Kaas method for the construction of N-substituted pyrrole derivatives **25** in 70–98% yields through the reaction between aryl-/alkyl-, sulfonyl-, and acylamines **24** and 2,5-dimethoxytetrahydrofuran (**2**) in the presence of 4 mol % ZrOCl_2_∙8H_2_O as a catalyst in water at 60 °C (Scheme 11a). Among many catalysts used for the synthesis of N-substituted pyrroles, Zr(IV) compounds, especially ZrOCl_2_∙8H_2_O have received more attention because of its high coordination ability, low toxicity, low cost and high activity. In addition, this procedure has many other advantages such as simple experimental work-up, easy availability of reagents, high to excellent yields, and environmental friendliness of the catalyst. To optimize the reaction conditions, the authors screened different solvents, catalyst loading, and temperature. The best result was obtained when the model reaction was run at 60 °C for 30 min in the presence of 5 mol % of ZrOCl_2_∙8H_2_O catalyst in water as a solvent. Among the different solvents used, water turned out to be the best solvent because it takes a short time and gives a higher yield of product compared to other organic solvents. In addition, the use of water as the green solvent for this conversion contributed more to the development of a green reaction. The proposed mechanism is depicted in Scheme 11b, in which the methoxy group of 2,5-DMTHF (**2**) is opened by the deprotection in acidic medium and forms an unstable intermediate that easily forms activated dialdehyde **B**. The amine **24** reacts with activated dialdehyde and provides the corresponding pyrrole **25** through the removal of water molecules.

**Scheme 12:**
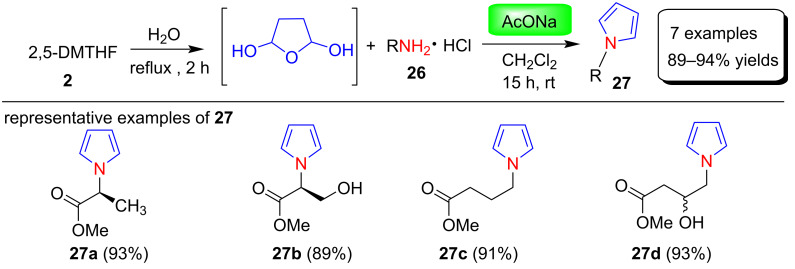
AcONa catalyzed synthesis of N-substituted pyrroles **27**.

Smith and co-workers [65] reported a modified one-pot, two-step Clauson–Kaas procedure for the synthesis of various acid- or heat-sensitive N-substituted pyrrole derivatives **27**. In this method, 2,5-dimethoxytetrahydrofuran undergoes mild hydrolysis in water to provide an activated species that reacts with various primary amines **26** in the presence of acetate buffer solution at ambient temperature to afford various N-substituted pyrroles **27** in 89–94% yields (Scheme 12). A major advantage of this protocol is that in the case of chiral amines, pyrrole formation proceeds without detectable epimerization.

**Scheme 13:**
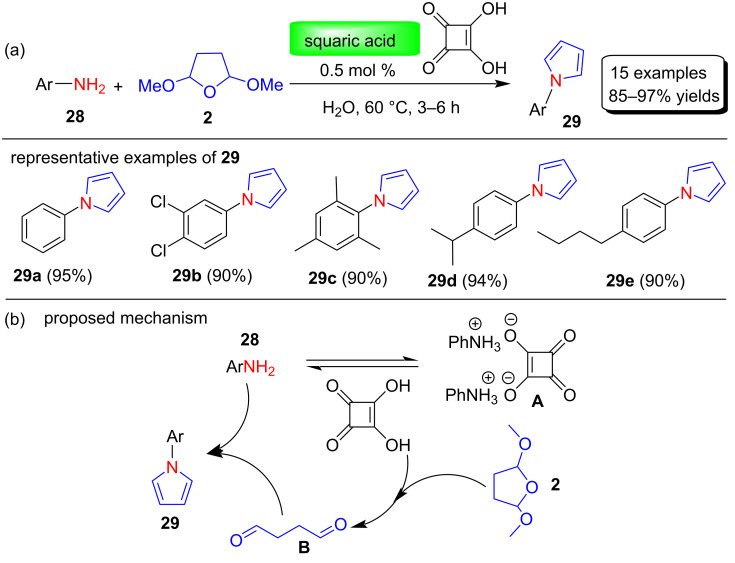
Squaric acid-catalyzed synthesis and proposed mechanism of N-substituted pyrroles **29**.

In 2013 Azizi et al. [66] have demonstrated a simple and environmentally friendly protocol for the synthesis of N-substituted pyrroles **29** in the yields of 85–97% using squaric acid as catalyst. In this reaction, various amines **28** and 2,5-DMTHF (**2**) were reacted in greener solvents like water, deep eutectic solvent (DEC) and polyethylene glycol (PEG) under thermal or ultrasonic irradiation (Scheme 13a).

The function of squaric acid as a catalyst was not clear, but the authors suggested that the Brønsted acidity of squaric acid affects the reactivity and selectivity of this process. The tentative mechanism of this protocol was proposed (Scheme 13b), in which a reversible acid–base reaction of aniline **28** with squaric acid afforded anilinium squarate salt **A**. Further, a catalytic amount of squaric acid hydrolyzes 2,5-dimethoxytetrahydrofuran (**2**) to give a 1,4-dicarbonyl compound **B** in water. Finally, *N*-phenylpyrrole **29** was obtained by condensation of activated 1,4-dicarbonyl compound with aniline.

**Figure 3:**
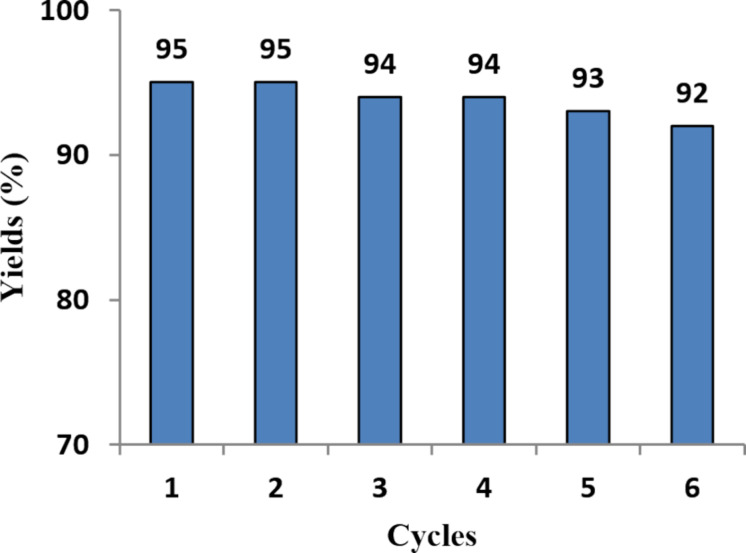
Reusability of catalyst γ-Fe_2_O_3_@SiO_2_-Sb-IL in six cycles.

**Scheme 14:**
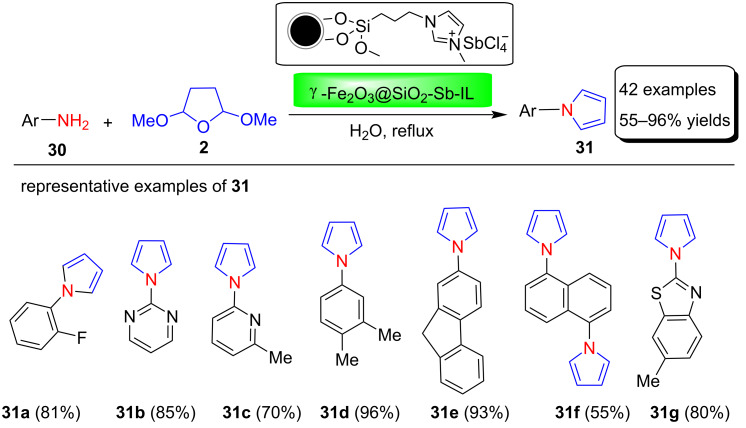
Magnetic nanoparticle-supported antimony catalyst used in the synthesis of N-substituted pyrroles **31**.

In 2013, Zhang and co-workers [67] reported the preparation of a new recyclable magnetic nanoparticle-supported antimony catalyst (γ-Fe_2_O_3_@SiO_2_-Sb-IL) and its application in the filtration-free, Clauson–Kaas synthesis of N-substituted pyrroles. This catalyst is fairly easy to make, air stable, and magnetically recoverable by simple magnetic decantation. In addition, the catalytic activity of the catalyst remains unaltered after six consecutive cycles (Figure 3). Using this catalyst, various nitrogen-substituted pyrrole derivatives **31** were synthesized in 55–96% yields through the reaction between various amines **30** and 2,5-dimethoxytetrahydrofuran (**2**) under aqueous conditions (Scheme 14). Among various solvents used to optimize the reaction conditions, H_2_O proved to be the best solvent for these transformations.

**Scheme 15:**
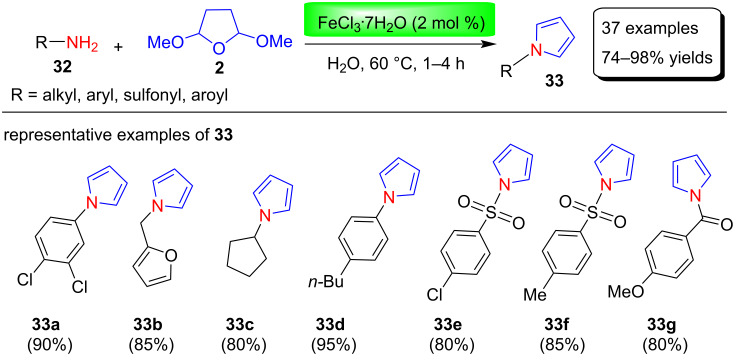
Iron(III) chloride-catalyzed synthesis of N-substituted pyrroles **33**.

Azizi and co-workers [68] described a one step, economical, and green method for the synthesis of N-substituted pyrroles using iron(III) chloride as a Lewis acid catalyst. These nitrogen-substituted pyrroles **33** were obtained in 74–98% yields by the reaction between various alkyl-, aryl-, sulfonyl- and aroylamines **32** with 2,5-DMTHF (**2**) in the presence of 2 mol % FeCl_3_**∙**7H_2_O as catalyst under H_2_O at 60 °C (Scheme 15). Among the different solvents used to optimize the reaction conditions, H_2_O turned out to be a better and greener solvent compared to other organic solvents (e.g., MeCN, C_6_H_6_, CH_2_Cl_2_, THF, EtOH, EtOAc).

**Scheme 16:**
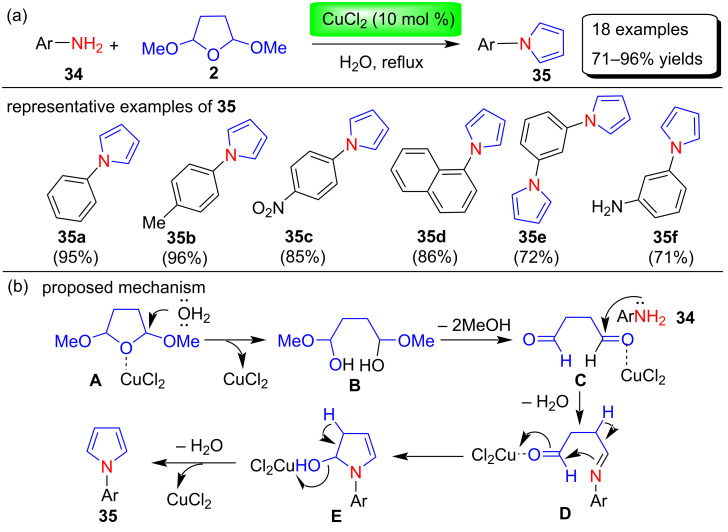
Copper-catalyzed Clauson–Kaas synthesis and mechanism of pyrroles **35**.

Deng et al. [69] brilliantly described an expedient copper-catalyzed Clauson–Kaas synthesis of a wide range of N-substituted pyrroles **35** in excellent yields of 71–96% by the reaction between various amines **34** and 2,5-dimethoxytetrahydrofuran (**2**) in water at reflux conditions (Scheme 16a). This protocol requires an easy workup, an inexpensive catalyst, mild reaction conditions, and simple operation in a more environmentally benign environment. Among various catalysts (CuO, CuSO_4_, Cu(OAc)_2_, CuF_2_, CuBr_2_, CuCl, CuI, Cu_2_O, CuCl_2_), solvents (THF, EtOAc, EtOH, CH_2_Cl_2_, CH_3_CN, H_2_O) and amount of catalyst loading (5, 10, 15 mol %) to optimize the reaction conditions, CuCl_2_ as the catalyst, H_2_O as solvent, and 10 mol % catalyst loading were chosen for the synthesis of various N-substituted pyrrole products. Interestingly, the Clauson–Kaas reaction of 2,5-DMTHF (**2**) with *m*-phenylenediamine or *p*-phenylenediamine proceed readily to afford the corresponding N-substituted monopyrroles and bispyrroles in good yields and high selectivity. To control the selectivity for the synthesis of mono- and bispyrrole-containing compounds, two different reaction conditions have been used. One equivalent of 2,5-DMTHF (**2**) was used for the synthesis of monopyrroles under reflux conditions for 2 hours, whereas two equivalents of 2,5-DMTHF were needed for the synthesis of bispyrrole under 8 hours reflux conditions. During this study, it was found that electron-donating groups on the phenyl ring of aromatic amines favor the formation of the corresponding product in excellent yields, whereas the electron-withdrawing groups of the aromatic amine slightly decreases the reactivity of the substrate and requires more time for the formation of products. The authors proposed a tentative mechanism for the formation of N-substituted pyrroles as shown in Scheme 16b. In the first step, intermediate **B** is formed by the hydrolysis of 2,5-DMTHF (**2**), which provide intermediate **C** after the removal of methanol in the presence of CuCl_2_. In the next steps, nucleophilic addition reaction of amines **34** with intermediate **C**, dehydration, and intramolecular aromatization affords N-substituted pyrroles **35**.

**Scheme 17:**
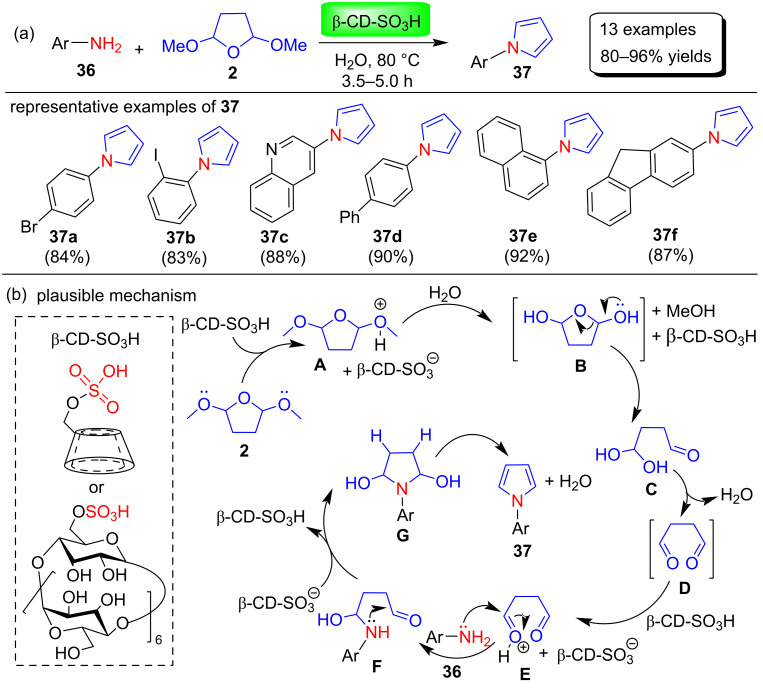
β-CD-SO_3_H-catalyzed synthesis and proposed mechanism of pyrroles **37**.

**Figure 4:**
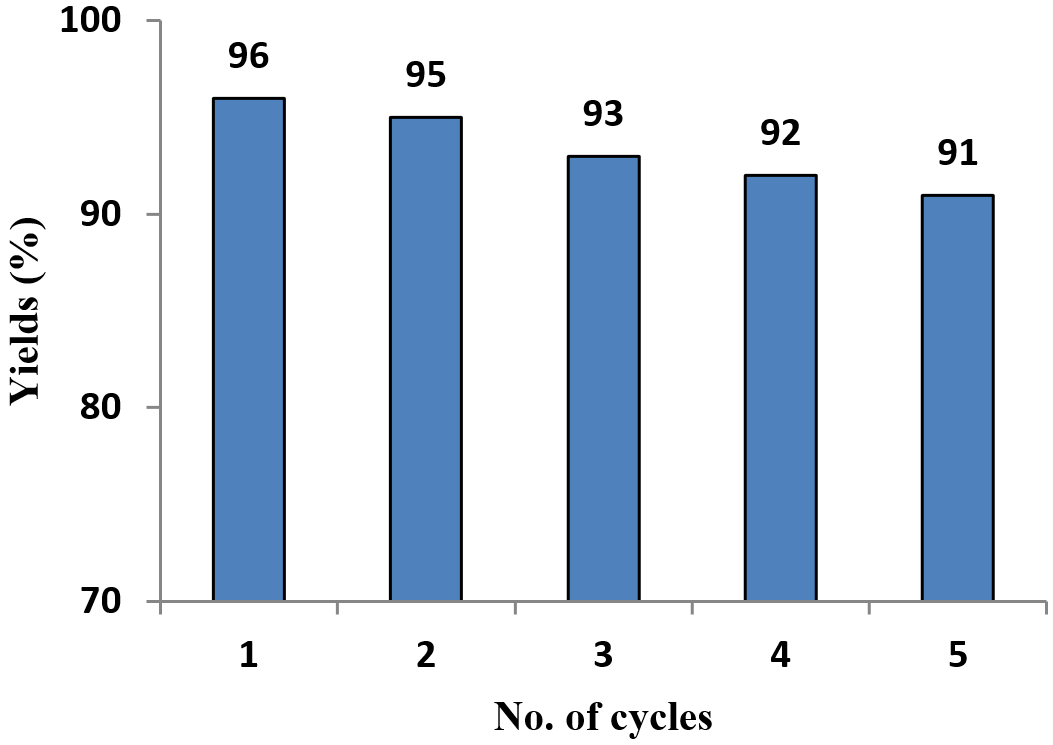
Recyclability of β-cyclodextrin-SO_3_H.

In 2018, Patil and Kumar [70] demonstrated a mild and environmentally friendly biomimetic Clauson–Kaas synthesis of N-substituted pyrroles **37** through the reaction between various arylamines **36** and 2,5-DMTHF (**2**) using a sustainable catalyst β-cyclodextrin-SO_3_H in the nontoxic green solvent H_2_O without the formation of side products (Scheme 17a). In this protocol, cyclodextrin (CD) plays a dual role as an acid catalyst as well as a phase-transfer agent that facilitates the smooth conversion of reactants into products. Most importantly, this catalyst can be easily recovered and used again for up to five cycles (Figure 4), with almost no effect on product yield and no loss of catalytic activity. Moreover, a plausible mechanism for the constructions of N-arylpyrroles is shown in Scheme 17b. In the first step, the acetal group of 2,5-DMTHF (**2**) is protonated by β-CD-SO_3_H to produce intermediate **A**. Intermediate **A** is nucleophilically attacked by a water molecule to form intermediate **B**. Further, dehydration and protonation leads to the formation of intermediate **E**. Nucleophilic attack of amine **36** on **E** then affords intermediate **F**. Finally, protonation, cyclization, and dehydration afford N-substituted pyrrole **37**. The authors also demonstrated the synthesis of polygonatine, an alkaloid natural product, using this protocol.

#### (2) Reactions under solvent-free conditions

**Scheme 18:**
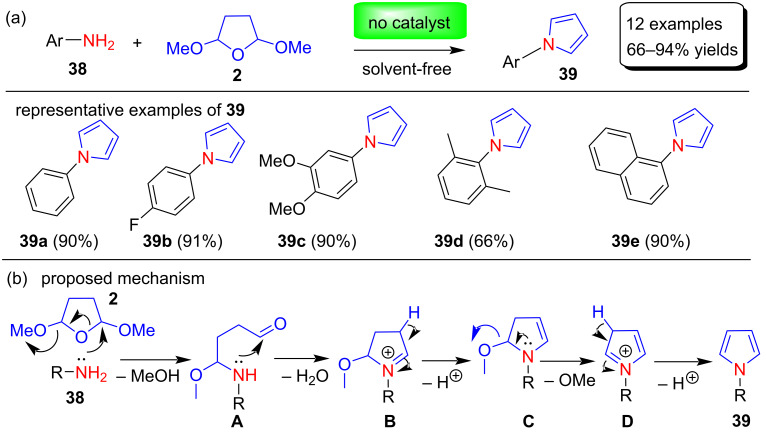
Solvent-free and catalyst-free synthesis and plausible mechanism of N-substituted pyrroles **39**.

In recent years, interest in organic reactions that do not use toxic organic solvents has increased due to environmental considerations. Ramesh et al. [71] in 2012 reported an efficient, economical, greener solvent-free process for synthesizing N-substituted pyrroles **39** with a yield of 66–94% by the reaction between different aromatic amines **38** and 2,5-dimethoxytetrahydrofuran (**2**) without using any catalyst or solvent (Scheme 18a). This method is also effective for aniline having electron withdrawing and electron donating substituents on the aromatic ring. However, this protocol did not produce a product with aliphatic amines and satirically hindered *o-*substituted anilines. A plausible mechanism for the formation of N-arylpyrroles is depicted in Scheme 18b.

**Scheme 19:**
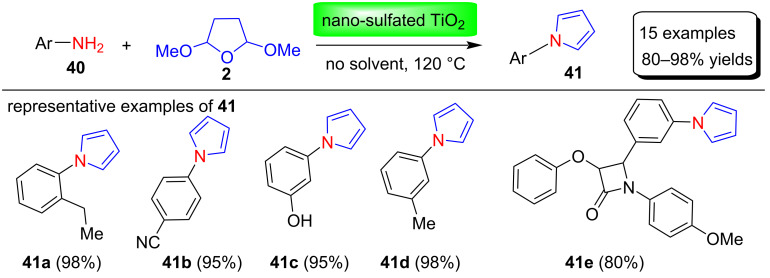
Nano-sulfated TiO_2_-catalyzed synthesis of N-substituted pyrroles **41**.

In 2014, Hosseini-Sarvari and co-workers [72] described a new and greener Clauson–Kaas method for the synthesis of N-substituted pyrroles **41** in 80–98 % yields by condensing 2,5-DMTHF (**2**) with amines **40** in the presence of the novel heterogeneous catalyst nano-sulfated TiO_2_ under solvent-free conditions (Scheme 19). This process has many advantages such as ease of operation, short reaction times, high to excellent product yields, and stable, readily available and green catalysts. Moreover, this protocol is also useful for the preparation of N-substituted compounds with β-lactam fragments.

**Figure 5:**
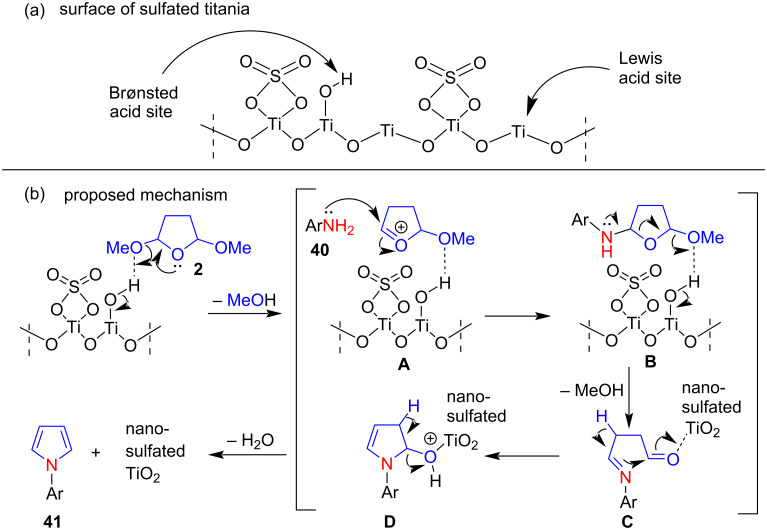
Plausible mechanism for the formation of N-substituted pyrroles catalyzed by nano-sulfated TiO_2_ catalyst.

The authors also proposed a mechanism for the synthesis of nano-sulfated titania-catalyzed N-substituted pyrroles as shown in Figure 5b. In general, both Lewis and Brønsted acid sites are present in sulfated metallic oxides, as shown in Figure 5a. The acidity of these Brønsted acid sites is increased by the presence of adjacent strong Lewis acid sites, and the acidity of these Lewis acid sites is due to the inductive effect of sulfate on the metallic cation. Therefore, this nano-sulfated titanium dioxide acts as a new type of Lewis acid catalyst. Intermediate **A** was first formed by reaction of the catalyst with 2,5-DMTHF (**2**). Further, a nucleophilic attack of amines **40** with intermediate **A**, MeOH removal, dehydration, and aromatization steps produce N-substituted pyrroles **41** in good to excellent yields.

**Scheme 20:**
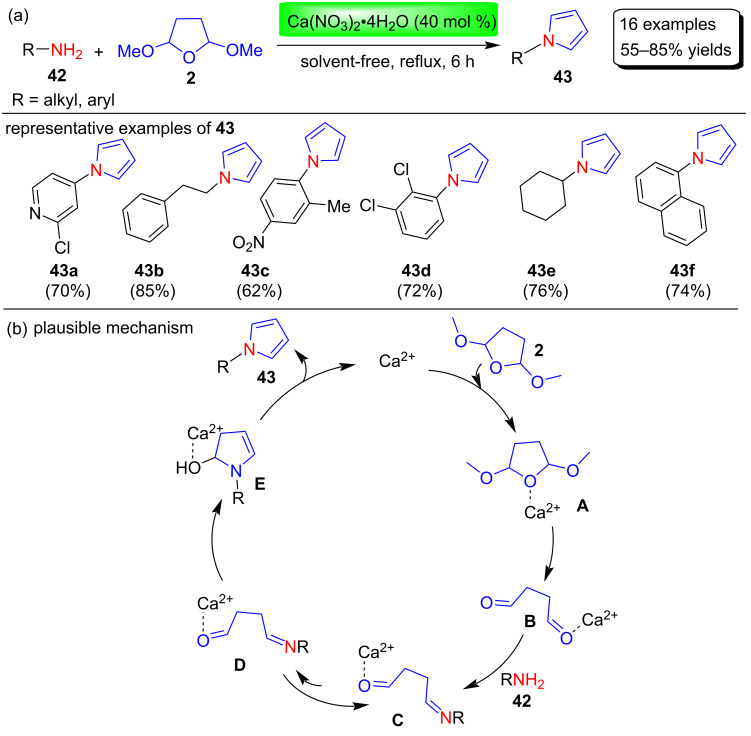
Copper nitrate-catalyzed Clauson–Kaas synthesis and mechanism of N-substituted pyrroles **43**.

In 2019, Wani et al. [73] used the alkaline-earth metal-based catalyst Ca(NO_3_)_2_∙4H_2_O for a mild, transition metal-free and greener synthesis of various N-substituted pyrroles **43** under solvent-free conditions (Scheme 20a). For the optimizations of the reaction conditions, various catalysts (Ca(NO_3_)_2_∙4H_2_O, UO_2_(NO_3_)_2_∙6H_2_O, Bi(NO_3_)_2_∙5H_2_O) and solvents (H_2_O, CH_3_CN, EtOH, DMSO, DMF, MeOH, CH_2_Cl_2_, no solvent) were investigated. As a result, 40 mol % Ca(NO_3_)_2_∙4H_2_O was selected as the best catalyst under solvent-free conditions and used to prepare 16 examples of various N-aryl-substituted pyrrole derivatives in yields of 55–85%. The authors also proposed a mechanism for the preparation of these N-substituted pyrroles as shown in Scheme 20b.

In this mechanism, calcium nitrate first activates the decomposition process of 2,5-DMTHF (**2**) from **A** to **B**. Further, a nucleophilic attack of amines **42** on **B** generates imine intermediate **C**. Finally, intramolecular nucleophilic attack by enamine **D** on the aldehyde group, dehydration and aromatization steps led to product **43** upon catalyst regeneration.

**Scheme 21:**
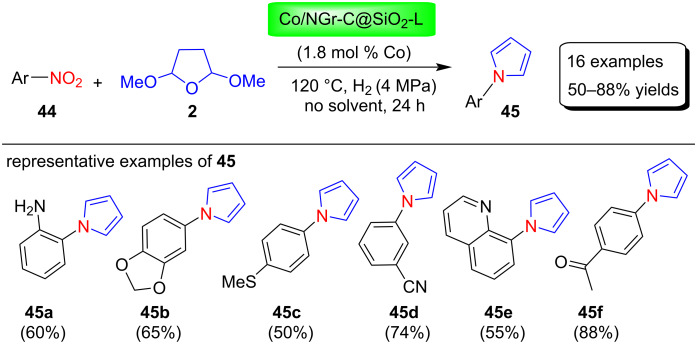
Synthesis of N-substituted pyrroles **45** by using Co catalyst Co/NGr-C@SiO_2_-L.

Recently, Ryabchuk et al. [74] used the 3d-metal cobalt catalyst Co/NGr-C@SiO_2_-L under solvent-free conditions to synthesize various N-aryl-substituted pyrroles **45** in 50–88% yields from the corresponding nitroarenes **44** via the Clauson–Kaas reaction involving benign reducing agents H_2_ or HCOOH or CO/H_2_O mixtures (Scheme 21). The main advantage of this heterogeneous Co catalyst is that it can be used up to 10 times without significant loss of activity and the active cobalt hydride species selectively reduces nitroarenes to their corresponding amines during pyrrole synthesis. This protocol has a high functional group tolerance and has been used to manufacture many major pharmaceutical compounds.

**Scheme 22:**
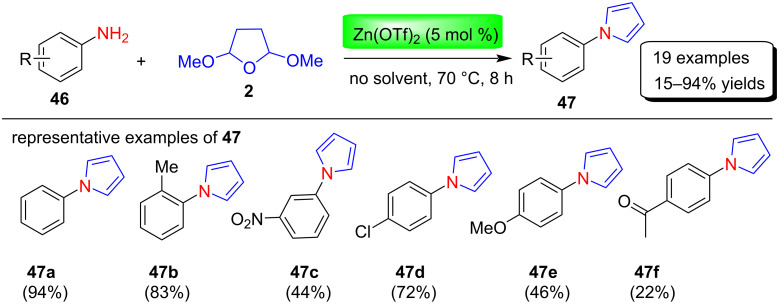
Zinc-catalyzed synthesis of *N*-arylpyrroles **47**.

Very recently, Afsina et al. [75] reported a solvent-free greener protocol for the synthesis of N-substituted pyrroles via the modified Clauson–Kaas method using a zinc catalyst. The synthesis of pyrrole derivatives **47** were achieved in moderate to excellent yields without using any co-catalyst, ligands or bases by stirring various aniline derivatives **46** with 2,5-dimethoxytetrahydrofuran (**2**) in the presence of 5 mol % of Zn(OTf)_2_ for 8 hours at 70 °C. Many zinc catalysts (Zn(OTf)_2_, Et_2_Zn, ZnI_2_, Zn powder, anhydrous ZnCl_2_, Zn(NO_3_)_2_⋅6H_2_O, ZnSO_4_⋅H_2_O, Zn(OAc)_2_⋅2H_2_O and ZnO), reaction temperature, time, and catalyst loading was screened for the optimization. After optimizations, 5 mol % Zn(OTf)_2_ as catalyst, reaction time for 8 h, and temperature of 70 °C were selected as optimal reaction conditions, and used for the preparations of 19 examples of N-aryl-substittuted pyrroles in 15–94% yields (Scheme 22).

**Scheme 23:**
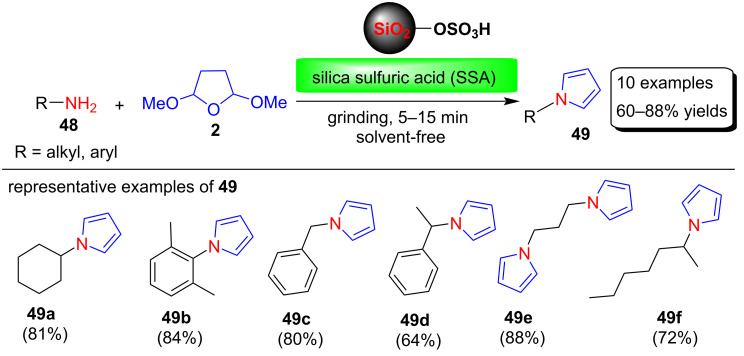
Silica sulfuric acid-catalyzed synthesis of pyrrole derivatives **49**.

In another report, Aydogan and co-workers demonstrated a silica sulfuric acid (SSA) catalyzed solvent-free Clauson–Kaas synthesis of N-substituted pyrrole in 60–80% yields in very short reaction times [76] (Scheme 23). To optimize the reaction conditions, several green protocols were used, among these sulfuric acid-immobilised on silica gel (SSA) catalyst under solvent-free conditions was chosen for the synthesis of these N-substituted pyrrole derivatives.

**Scheme 24:**
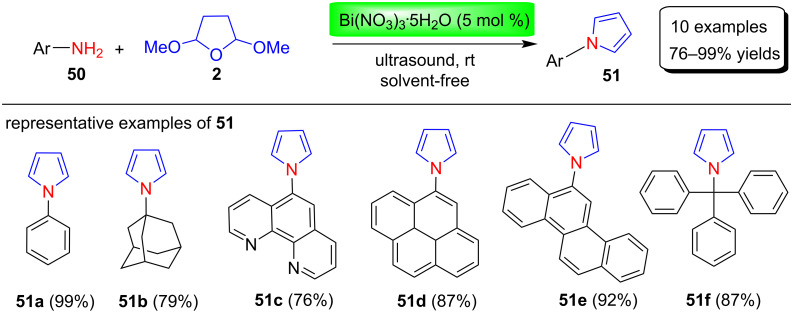
Bismuth nitrate-catalyzed synthesis of pyrroles **51**.

In 2012, Bandyopadhyay et al. [77] reported the solvent-free and eco-friendly synthesis of various N-substituted pyrroles **51** in excellent 76–99% yields using bismuth nitrate as catalyst (Scheme 24). In this reaction, various aliphatic, aromatic, polyaromatic and heteropolyaromatic amines **50** were reacted with 2,5-DMTHF (**2**) in 5 mol % Bi(NO_3_)_3_·5H_2_O in solvent-free conditions under ultrasound irradiation at room temperature. The main advantages of this procedure are its simple isolation process, excellent product yield without using expensive or sensitive solvents and reagents/instruments. Some of these synthesized N-substituted pyrrole derivatives were evaluated for in vitro cytotoxicity for various cancer cell lines.

**Scheme 25:**
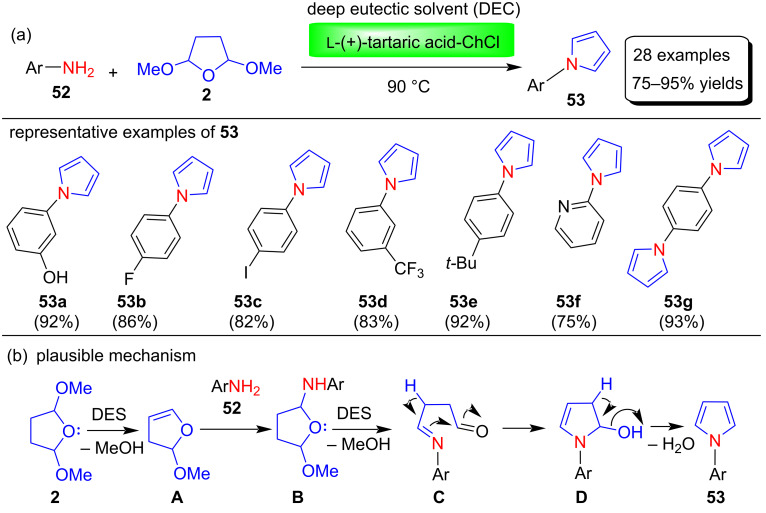
L-(+)-tartaric acid-choline chloride-catalyzed Clauson–Kaas synthesis and plausible mechanism of pyrroles **53**.

In 2014, Wang et al. [78] demonstrated a modified Clauson–Kaas reaction to synthesize various N-substituted pyrroles **53** in 75–95% yields by reacting various aromatic amines **52** with 2,5-DMTHF (**2**) under a L-(+)-tartaric acid-choline chloride based deep eutectic solvent as green medium (Scheme 25a). Deep eutectic solvents (DESs) are mainly synthesized by mixing quaternary ammonium salts and hydrogen-bond donors. In this study, various combinations of salts and hydrogen bond donating groups were investigated. Among these, the DES prepared by mixing quaternary ammonium salts choline chloride (ChCl) and the hydrogen-bond donor L-(+)-tartaric acid gives the best result. Moreover, L-(+)-tartaric acid–ChCl acts as both solvent and catalyst for pyrrole synthesis. This process has many advantages because it is a metal-free, easy to use, and environmentally friendly method that also gives good product yields with a wide range of substrates. The deep eutectic solvent used in this protocol is cheap, reusable, non-toxic, and biodegradable. In this study, it was found that aniline bearing an electron-donating group produced slightly higher yields than aromatic amines with an electron-withdrawing group. The authors also proposed a tentative mechanism for this reaction, in which intermediate **A** was first formed by deprotection of 2,5-DMTHF (**2**), and then **A** reacted with amines **52** to produce N-substituted pyrroles **53** by nucleophilic addition, subsequent expulsion of methanol, followed by dehydration and aromatization steps (Scheme 25b). The authors suggested that the acidity of DESs may play an important role in the removal of the methoxy groups, and the hydrogen-bonding interaction between DES and the amino group enhance the nucleophlicity of the amines.

#### (3) Microwave-assisted reactions

Microwave-assisted heating offers a number of advantages over conventional heating, such as greater precision, excellent product yields, and very rapid reaction. This section describes the modified Clauson–Kaas synthesis of different pyrroles in water, solvent-free conditions, and in different organic solvents.

**Scheme 26:**
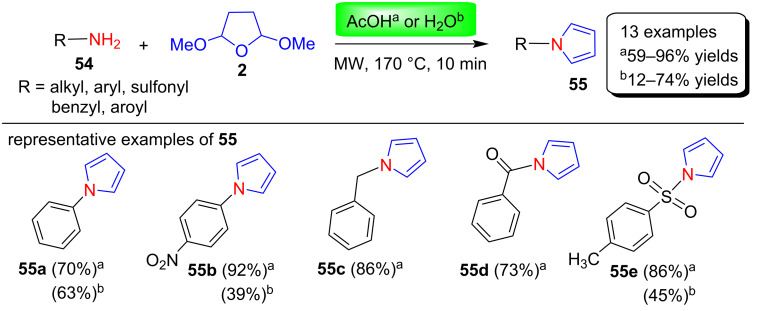
Microwave-assisted synthesis of N-substituted pyrroles **55** in AcOH or water.

**(i) Microwave-assisted reactions in water:** In 2009, Ketcha and colleagues [79] nicely utilized the Clauson–Kaas reaction for the environmentally friendly synthesis of various N-substituted pyrroles **55** through the microwave-assisted reaction of various primary amines **54** with 2,5-dimethoxytetrahydrofuran (**2**). In this protocol, without the use of any additional catalyst, the products were obtained in 12–74% yields in water and 59–96% yields in acetic acid, respectively (Scheme 26). This reaction has been successfully used for common amines using acetic acid and water, but benzylamines and benzamides show no reaction under aqueous conditions. In the case of amino acid ester hydrochlorides, the reaction to give pyrrole can be carried out without the need for the two-phase conditions by lowering the temperature from 170 °C to 120 °C.

**Scheme 27:**
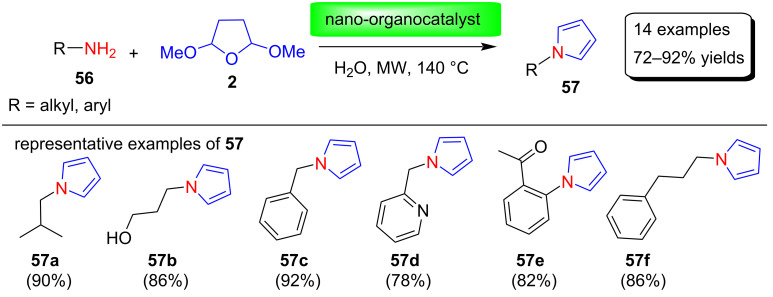
Synthesis of pyrrole derivatives **57** using a nano-organocatalyst.

**Figure 6:**
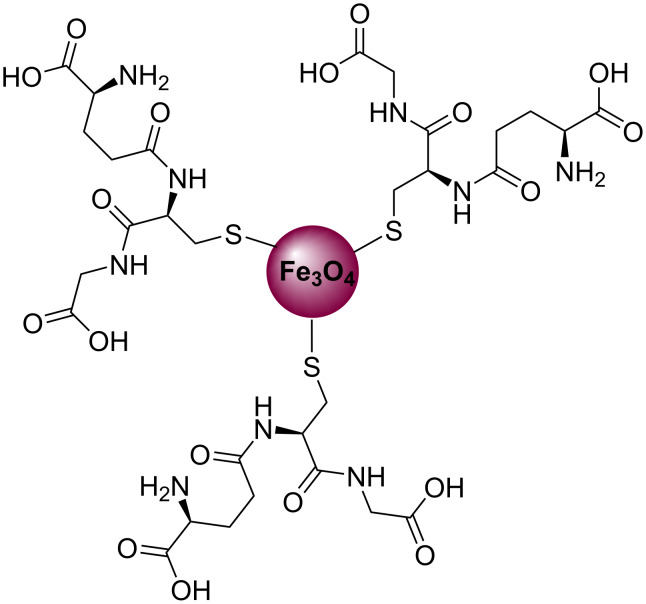
Nano-ferric supported glutathione organocatalyst.

Varma and co-workers [80–81] reported the synthesis of various N-substituted pyrrole derivatives **57** in good yields using a nano-ferric-supported glutathione organocatalyst (Scheme 27, Figure 6). This organoccatalyst was prepared by a post-functionalization method using a benign and naturally occurring glutathione and magnetic ferrite nanoparticles by sonication in water at room temperature. Furthermore, using this organocatalyst, various N-substituted pyrroles were prepared by reacting various amines **56** with 2,5-DMTHF (**2**) in water at 140 °C under microwave conditions. The main advantages of this protocol are the environmentally friendly reaction medium, recoverable catalysts, high yields, and the absence of toxic solvents.

**Scheme 28:**
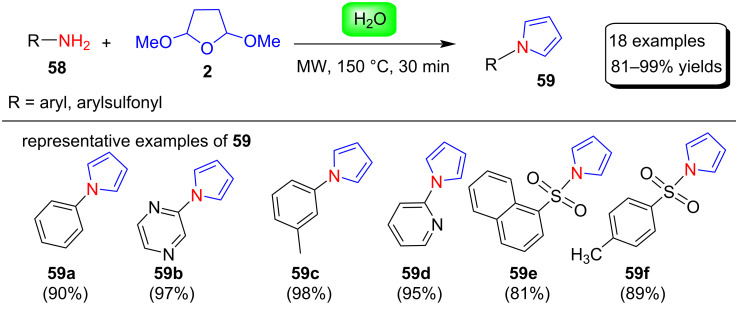
Microwave-assisted synthesis of N-substituted pyrroles **59** in water.

In 2009, Wilson et al. [82] described a simple and efficient synthesis of N-substituted pyrrole derivatives without the use of any catalysts under microwave conditions. In this procedure, various arylsulfonamides or anilines **58** are heated with 2,5-dimethoxytetrahydrofuran (**2**) in H_2_O under microwave irradiation conditions to produce the corresponding N-substituted pyrrole derivatives **59** in 81–99% yields (Scheme 28). A variety of amine nucleophiles were examined in this method. As a result, it was found that nucleophiles with low p*K*_a_ values, such as sulfonamides, reacted very well, while reactions with nucleophiles with high p*K*_a_ values, such as benzylamine, did not work well. Compared with other classical methods, this protocol is easy to use, cheap and environmentally friendly.

**Scheme 29:**
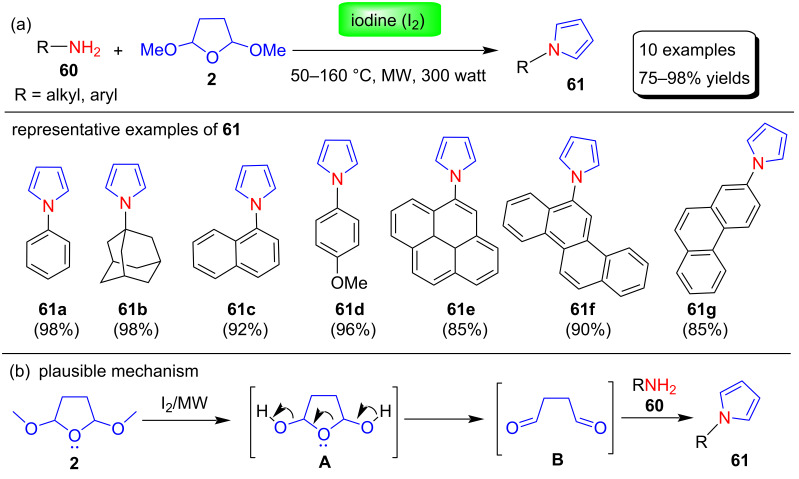
Iodine-catalyzed synthesis and proposed mechanism of pyrroles **61**.

**(ii) Microwave-assisted reactions under solvent-free conditions:** Banik et al. [83] described the synthesis of diverse N-substituted pyrroles using a microwave-assisted and iodine-catalyzed protocol. These pyrrole derivatives were prepared in 75–98% yields by reacting various amines **60** and 2,5-DMTHF **2** under solvent-free conditions in the presence of 5 mol % molecular iodine as catalyst (Scheme 29a). These synthesized products were tested against various cancer cells in vitro. In the proposed mechanism, deprotection of the methoxy group of 2,5-DMTHF (**2**) in the presence of iodine under MW irradiation afford intermediate **A**, which is converted to dialdehyde **B**. Finally, N-substituted pyrroles **61** are produced by nucleophilic addition of amines with dialdehyde, followed by dehydration and aromatization steps (Scheme 29b).

**Scheme 30:**
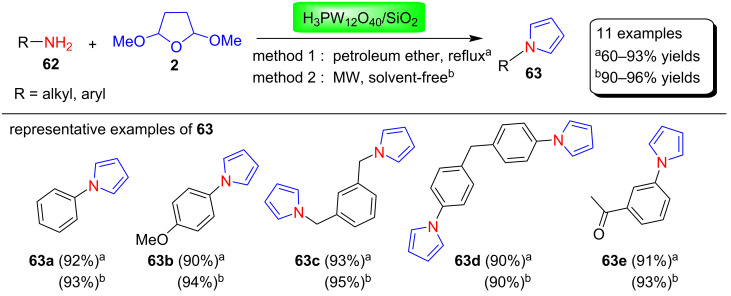
H_3_PW_12_O_40_/SiO_2_-catalyzed synthesis of N-substituted pyrroles **63**.

Jafari et al. [84] used the heterogeneous catalyst H_3_PW_12_O_40_/SiO_2_ for the Clauson–Kaas synthesis of N-substituted pyrrole derivatives **63** (Scheme 30) by the reaction of amines **62** with 2,5-dimethoxytetrahydrofuran (**2**) in petroleum ether at reflux conditions in 60–93% yields (method 1) and MW-assisted solvent-free conditions in 90–96% yields (method 2). The optimization of the reaction conditions was performed in search of suitable conditions for this condensation reaction. Various acid catalysts (SiO_2_, HPA, HPA/SiO_2_, catalyst loadings (1 mol %, 2 mol %, 2.5 mol %, 0.3 g), solvent-systems (petroleum ether 40/60, toluene, *n*-hexane, acetonitrile) and reaction conditions (room temperature, 60 °C, reflux, and MW (power 5, 8 or 10), were studied. Among these, the optimized reaction conditions for method 1 are 2.5 mol % HPA/SiO_2_ as catalyst in refluxing petroleum ether, whereas the optimized conditions for method 2 are 2.5 mol % HPA/SiO_2_ as catalyst, solventless under MW irradiation. The main advantages of this protocol is its greener, eco-friendly, stable and reusable heterogeneous catalyst, smooth and selective reaction under solvent-free conditions, excellent yields of products, easy work-up and simple handling of reaction.

**Scheme 31:**
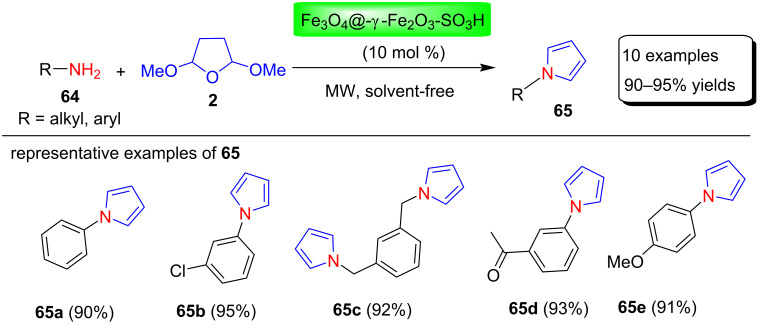
Fe_3_O_4_@-γ-Fe_2_O_3_-SO_3_H-catalyzed synthesis of pyrroles **65**.

Mahmoudi and Jafari [85] reported the preparation of a new on magnetic nanoparticles sulfonic acid-supported catalyst with maghemite coating as a magnetically recyclable catalyst Fe_3_O_4_@-γ-Fe_2_O_3_-SO_3_H. This heterogeneous catalyst is used for the synthesis of N-substituted pyrrole derivatives **65** in 90–95% yields using a solvent-free reaction of amines **64** with 2,5-DMTHF (**2**) under microwave irradiation conditions (Scheme 31). This method has many advantages, including simpler, less expensive, and faster catalyst preparation, easy removal of the catalyst from the reaction mixture, and use of the catalyst for up to nine consecutive runs without loss of catalytic activity.

**Scheme 32:**
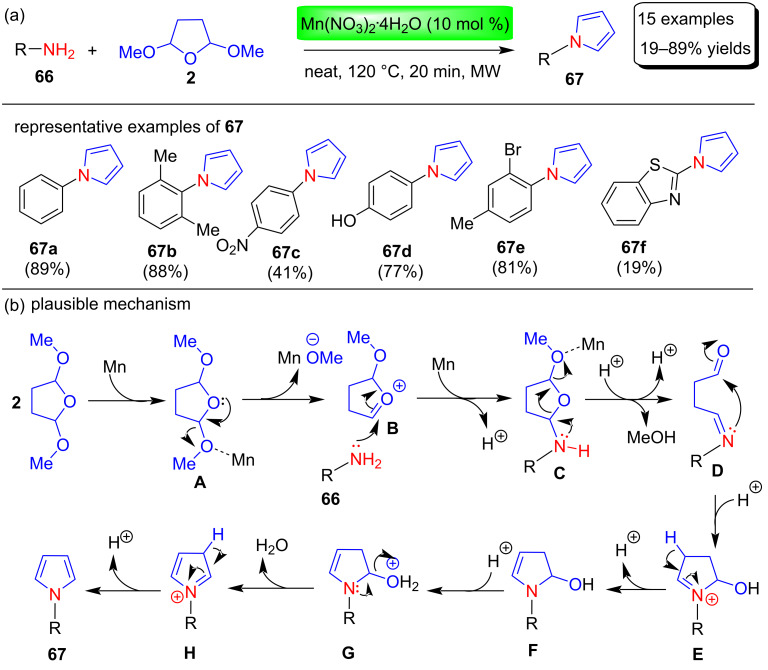
Mn(NO_3_)_2_·4H_2_O-catalyzed synthesis and proposed mechanism of pyrroles **67**.

Recently, Rohit et al. [86] described a modified Clauson–Kaas synthesis of N-substituted pyrrole with up to 89% yields by microwave irradiation at 120 °C in neat conditions by reacting amines **66** with 2,5-DMTHF (**2**) in the presence of Mn(II) catalyst Mn(NO_3_)_2_.4H_2_O (Scheme 32a). Numerous Mn catalysts (e.g., MnCl_2_∙4H_2_O, MnSO_4_∙H_2_O, Mn(NO_3_)_2_∙4H_2_O, Mn(OAc)_2_∙4H_2_O, MnBr(CO)_5_, MnO_2_), catalyst loading (e.g., 0, 5, 10, 20 mol %), reaction temperatures (90–130 °C) and reaction times (e.g., 10, 20, 25 min) were investigated in order to optimize the reaction conditions. Among these, 10 mol % Mn(NO_3_)_2∙_4H_2_O was selected as the best catalyst for the synthesis of the pyrrole derivative **67**, with yields of 19–89% under MW in solvent-free conditions at 120 °C for 20 min. According to the results of this study, it was discovered that amines with electron-donating substituents produce higher yields of the final product than those with electron-withdrawing substituents. The authors also put forth a plausible mechanism for this protocol, in which Mn first coordinates with an OMe group of 2,5-DMTHF (**2**) and helps in the removal of the methoxy group to yield intermediate **B**. The electron-deficient carbon of intermediate **B** is then attacked by the amine in a nucleophilic reaction to produce intermediate **C**, which is then followed by the removal of the second OMe group to produce intermediate **D**. Further, the lone pair of N attacks the carbonyl carbon to form the 5-membered ring **E** bearing the iminium ion. Finally, N-substituted amines **67** were obtained after deprotonation/protonation, dehydration, and aromatization steps as shown in Scheme 32b.

**Scheme 33:**
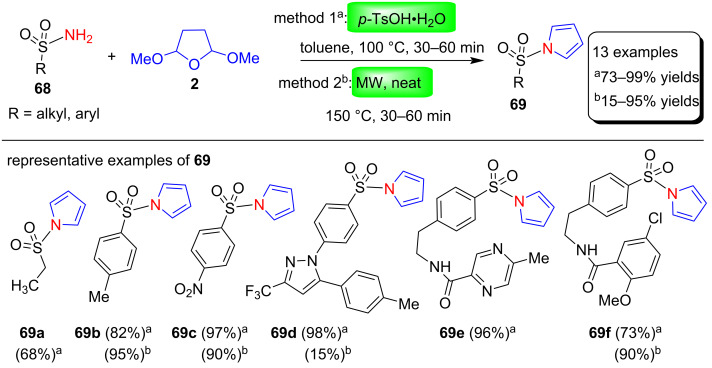
*p*-TsOH∙H_2_O-catalyzed (method 1) and MW-assisted (method 2) synthesis of *N*-sulfonylpyrroles **69**.

In another report, Ozaki et al. [87] used the Clauson–Kaas approach to synthesize sulfonic acids/sulfonates from various primary sulfonamides via sulfonylpyrroles. First, various sulfonylpyrroles **69** were prepared from primary sulfonamides **68** by reaction with 2,5-DMTHF (**2**) using two methods as shown in Scheme 33. In method 1, amides **68** and **2** were heated in toluene at 100 °C for 30–60 min in the presence of 5 mol % *p*-TsOH∙H_2_O. In contrast, method 2 involves a greener protocol in which amides **68** and **2** are reacted under microwave condition at 150 °C for 30–60 min without the use of additives or solvents. Furthermore, these sulfonylpyrroles were converted to the corresponding sulfonic acid/sulfonates. Using this method, various arylsulfonamides, alkylsulfonamides and diverse drug molecules bearing sulfonamides were easily converted to sulfonylpyrroles and then sulfonic acids. This cheap and easily accessible protocol may be useful in pharmaceutical and industrial applications.

**Scheme 34:**
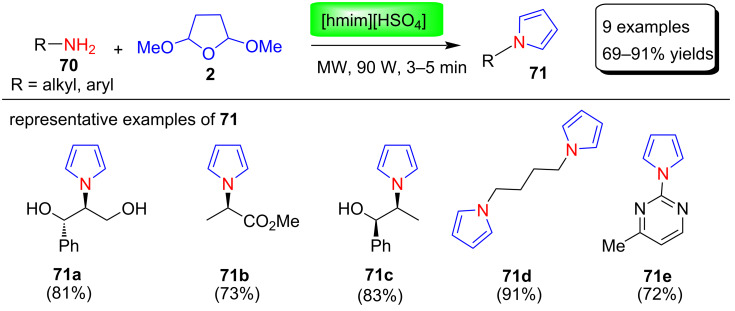
([hmim][HSO_4_]-catalyzed Clauson–Kaas synthesis of pyrroles **71**.

Aydogan and Yolacan [88] reported the Clauson–Kaas reaction for the preparation of acidic ionic liquid 1-hexyl-3-methylimidazolium hydrogen sulfate ([hmim][HSO_4_])-catalyzed N-substituted pyrrole derivatives **71** under microwave irradiation (Scheme 34). To optimize of reaction conditions, the catalytic activity of various acids and ionic liquids such as [hmim][HSO_4_], CH_3_COOH, [hmim][H_2_PO_4_], and [bmim][BF_4_] was investigated. Among these, [hmim][HSO_4_] proved to be the best catalyst providing higher yields in shorter time. This protocol provides a greener, environmental friendly, and a cleaner route for the synthesis of acid-sensitive pyrrole derivatives without decomposition in a short time.

**Scheme 35:**
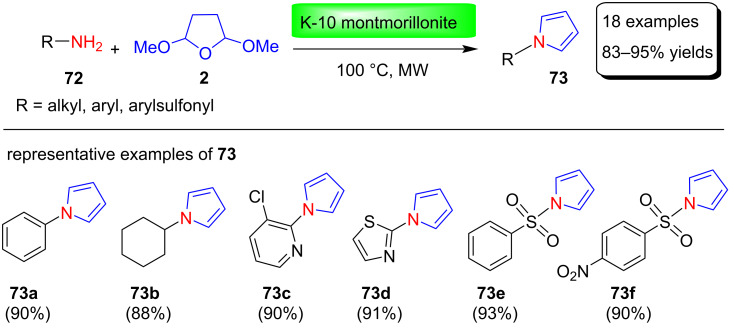
Synthesis of N-substituted pyrroles **73** using K-10 montmorillonite catalyst.

Abid et al. [89] described the synthesis of N-substituted pyrrole derivatives in good yields using K-10 montmorillonite as an effective solid acid catalyst under microwave irradiation. K-10 monomorillonite is a widely used solid acid catalyst, which features strong acidity, larger surface area and high stability. Pyrrole derivatives **73** were prepared in 83–95 % yields by condensation reactions between various alkyl-, aryl-, heteroaryl-, and sulfonylamines **72** and 2,5-dimethoxytetrahydrofuran (**2**) under MW conditions at 100 °C (Scheme 35). This solvent-free protocol has many advantages, including better selectivity and excellent product yields, mild reaction conditions, and ease of product isolation.

**Scheme 36:**
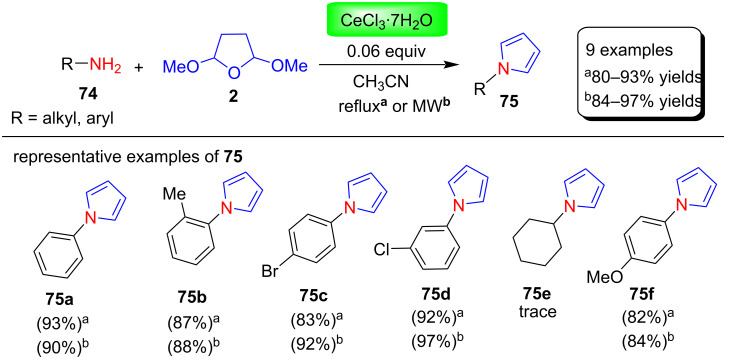
CeCl_3_∙7H_2_O-catalyzed Clauson–Kaas synthesis of pyrroles **75**.

**(iii) Microwave-assisted reactions under organic solvents:** Silveira and co-workers [90] developed the Clauson–Kaas synthesis of N-substituted pyrroles catalyzed by CeCl_3_∙7H_2_O. In this reaction, *N*-arylpyrroles **75** were synthesized from various aniline derivatives **74** by the reaction with 2,5-dimethoxytetrahydrofuran (**2**) in both microwave irradiation and conventional heating in acetonitrile (Scheme 36). Furthermore, these *N*-arylpyrroles were converted to the corresponding thiocyanated derivatives and then to sulfur-containing heterocycles. Moreover, this procedure provides N-substituted pyrroles from various aromatic amines in good to excellent yields under both reflux and MW conditions. In contrast, aliphatic amines such as cyclohexylamine and benzylamine give only traces of products even under prolonged reflux or MW irradiation times. In addition, it was also found that aniline bearing electron-donating substituents are less reactive and take more time to complete the reactions.

**Scheme 37:**
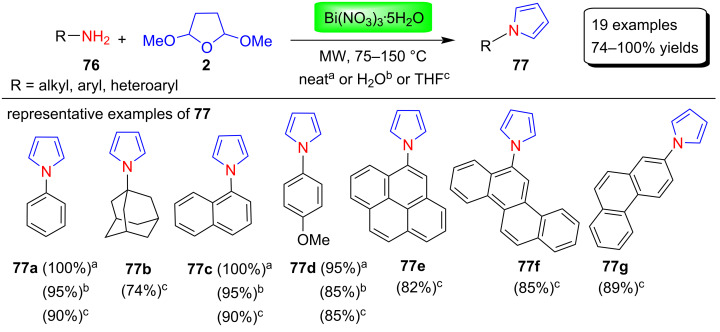
Synthesis of N-substituted pyrroles **77** using Bi(NO_3_)_3_∙5H_2_O.

Rivera et al. [91] synthesized N-substituted pyrrole derivatives **77** in good to excellent yields using a microwave-assisted and bismuth nitrate Bi(NO_3_)_3_∙5H_2_O-catalyzed reaction between various amines **76** and 2,5-dimethoxytetrahydrofuran (**2**) in neat, H_2_O or THF (Scheme 37). This approach provides different pyrrole derivatives without the use of sensitive or expensive reagents and also has numerous applications in other areas of research. In this study, it was discovered that amines with less nucleophilicity also produced good yields of the product. In contrast, for the reaction with polyaromatic amines, THF was found to be the best solvent because these compounds are insoluble in water and provides poor yields of the product in solvent-free conditions.

**Scheme 38:**
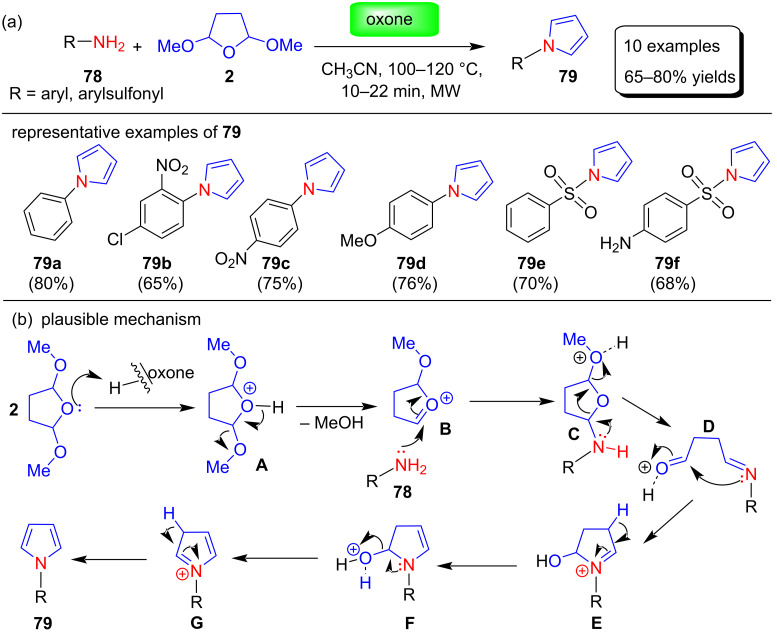
Oxone-catalyzed synthesis and proposed mechanism of N-substituted pyrroles **79**.

Gullapelli et al. [92] described the Clauson–Kaas synthesis of *N*-arylpyrroles **79** under microwave irradiation using oxone (2KHSO_5_∙KHSO_4_∙K_2_SO_4_) as a catalyst (Scheme 38a). Many solvents (EtOH, CH_3_CN, THF, DMF, H_2_O, neat), reaction times (10–22 minutes), and amount of oxone were investigated in order to stabilize the best reaction conditions. Among these, CH_3_CN as the best solvent system, 0.09 g of oxone, and a temperature of 110 ± 10 °C were chosen as optimized reaction conditions and used for the synthesis of *N*-arylpyrroles from various amines **78** via the reaction with 2,5-dimethoxytetrahydrofuran (**2**). Oxone is a mild, inexpensive, nontoxic, stable, and transition-metal-free catalyst that is very easy to handle during this transformation and provided high yields of the product. The authors also proposed a mechanism for this protocol as shown in Scheme 38b.

## Conclusion

The synthesis of pyrrole derivatives has piqued the interest of many synthetic researchers as they offer promising applications in pharmaceutical, polymer, and natural product chemistry. The introduction of a pyrrole moiety into a molecule alters its diverse properties and increases the possibility for further functionalization. The first section of this review successfully describes the use of the Clauson–Kaas reaction for the synthesis of N-substituted pyrroles using conventional methods such as Brønsted acid and Lewis acid-catalyzed reactions under heating conditions. However, due to environmental concerns, the second part of this review focuses on greener Clauson–Kaas reaction protocols. Various Brønsted acids, Lewis acids, transition metal catalysts, and organocatalysts have been used in water as a green solvent, under solvent-free conditions or in microwave-assisted reactions to synthesize various N-substituted pyrrole derivatives. Because of the high functional group tolerance of this method, various primary aliphatic/aromatic amines, benzamides, and sulfonamides have been used successfully as substrates in these transformations. It was discovered that, in general, arylamines with electron-withdrawing groups deactivated the arylamines and produced the corresponding pyrroles in only modest yields, whereas arylamines with electron-donating groups reacted much faster and produced the pyrroles in excellent yields. In addition, reactions of sterically hindered anilines with 2,5-dimethoxytetrahydrofuran give moderate yields, and reactions with less sterically hindered anilines give good yields of products. The Clauson–Kaas method for the synthesis of pyrroles has a wide range of applications, including the synthesis of various pharmaceutically active molecules, polymer synthesis, total synthesis of natural products, porphyrin functionalization, and the synthesis of various pyrrole-containing heterocyclic molecules. We believe that this review will be useful and will encourage researchers to apply the modified Clauson–Kaas reaction to different areas of chemistry.

**Table 1:** List of abbreviations.

Abbreviation	Name

AcOH	acetic acid
β-CD	β-cyclodextrin
Co-NP	cobalt nanoparticle
DES	deep eutectic solvent
DMAP	4-(dimethylamino)pyridine
DMF	*N,N*-dimethylformamide
2,5-DMTHF	2,5-dimethoxytetrahydrofuran
EtOH	ethanol
[Hmim][HSO_4_]	1-methylimidazolium hydrogen sulfate
HPA	hydroxypropyl acrylate
MeCN	acetonitrile
MW	microwave
NaOAc	sodium acetate
OPV	organic photovoltic
PEG	polyethylene glycol
*p*-TsOH	*p*-toluenesulfonic acid
SRR	structure–reactivity relationship
SSA	silica sulfuric acid
TEA	triethylamine
TfOH	trifluoromethanesulfonic acid
THF	tetrahydrofuran
